# Fas/FasL mediates NF-κBp65/PUMA-modulated hepatocytes apoptosis via autophagy to drive liver fibrosis

**DOI:** 10.1038/s41419-021-03749-x

**Published:** 2021-05-12

**Authors:** Siwei Tan, Xianzhi Liu, Lingjun Chen, Xiaoqin Wu, Li Tao, Xuemei Pan, Shuyan Tan, Huiling Liu, Jie Jiang, Bin Wu

**Affiliations:** 1 Department of Gastroenterology, the Third Affiliated Hospital of Sun Yat-Sen University, Guangzhou, Guangdong Province 510630 China; 2 Guangdong Provincial Key Laboratory of Liver Disease Research, Guangzhou, Guangdong Province 510630 China

**Keywords:** Apoptosis, Extracellular signalling molecules

## Abstract

Fas/Fas ligand (FasL)-mediated cell apoptosis involves a variety of physiological and pathological processes including chronic hepatic diseases, and hepatocytes apoptosis contributes to the development of liver fibrosis following various causes. However, the mechanism of the Fas/FasL signaling and hepatocytes apoptosis in liver fibrogenesis remains unclear. The Fas/FasL signaling and hepatocytes apoptosis in liver samples from both human sections and mouse models were investigated. *NF-κBp65* wild-type mice (*p65*^*f/f*^), hepatocytes specific *NF-κBp65* deletion mice (*p65*Δhepa), p53-upregulated modulator of apoptosis (PUMA) wild-type (*PUMA*-WT) and *PUMA* knockout (*PUMA*-KO) littermate models, and primary hepatic stellate cells (HSCs) were also used. The mechanism underlying Fas/FasL-regulated hepatocytes apoptosis to drive HSCs activation in fibrosis was further analyzed. We found Fas/FasL promoted PUMA-mediated hepatocytes apoptosis via regulating autophagy signaling and NF-κBp65 phosphorylation, while inhibition of autophagy or *PUMA* deficiency attenuated Fas/FasL-modulated hepatocytes apoptosis and liver fibrosis. Furthermore, NF-κBp65 in hepatocytes repressed PUMA-mediated hepatocytes apoptosis via regulating the Bcl-2 family, while *NF-κBp65* deficiency in hepatocytes promoted PUMA-mediated hepatocytes apoptosis and enhanced apoptosis-linked inflammatory response, which contributed to the activation of HSCs and liver fibrogenesis. These results suggest that Fas/FasL contributes to NF-κBp65/PUMA-modulated hepatocytes apoptosis via autophagy to enhance liver fibrogenesis, and this network could be a potential therapeutic target for liver fibrosis.

## Introduction

Liver fibrosis represents one of the major consequences of morbidity and mortality worldwide^1,2^, and the activation of hepatic stellate cells (HSCs), which is regulated by multiple cell populations or soluble mediators, is the major source of extracellular matrix substances (ECM)^3–5^. Hepatocytes are the major parenchymal cells of the liver and essential for maintaining the function and organization of the liver^6^. It’s widely accepted that the progression of hepatic fibrosis is associated with considerable injury and loss of hepatocytes, which may present as a main inflammatory stimulus for HSCs activation^7,8^. The apoptotic cells could release the nucleotides ATP and UTP, which bind to purinergic receptors (especially the P2Y_2_ receptor) on macrophages and HSCs, leading to their activation^9,10^. As a classic apoptosis modulator, Fas, following Fas ligand (FasL) engagement, leads to the recruitment of Fas-associated proteins having death domains and the initiators into a death-inducing signaling complex (DISC) to cause apoptosis^11–13^, and the Fas/FasL has been demonstrated to participate in the pathological processes of hepatic fibrosis/cirrhosis, acute/chronic hepatitis and hepatocarcinoma^14–16^.

Autophagy is an evolutionarily conserved and catabolic process that targets cytosolic material, organelles and long-lived proteins to lysosomes to be degraded for survival, development, differentiation, and homeostasis^17–19^. Hepatocytes have been revealed to own a high level of autophagic flux because of their increased abundance of lysosomes and lysosomal enzymes, and the enhanced autophagy could modulate the progression to hepatocytes death^20^, and our previous study has suggested autophagy was required for liver fibrogenesis^21^. Nuclear factor-κB (NF-κB), as a ubiquitous and inducible transcription factor responsible for mediating the expression of a large number of genes involved in differentiation, apoptosis, and proliferation^22^, and NF-κBp65, as the main functional element, involves in various physiological and pathological events and influences the survival of hepatocytes and activation of HSCs^23^. Activation of NF-κB in non-parenchymal cells promotes inflammation, fibrosis, and hepatocarcinogenesis in the liver, whereas suppression of NF-κB in parenchymal cells enhances hepatocarcinogenesis in some cases and retards hepatocarcinogenesis in others^10,24^. The activation of NF-κB in HSCs appears to promote hepatic fibrosis via modulating fibrogenic effects and anti-apoptotic effects^9^. While decreased or absent NF-κB activity in hepatocytes might lead to subsequent fibrosis by regulating hepatocytes injury and the primary trigger of fibrogenic responses in liver^23–26^.

Considerable death and loss of hepatocytes induced by different etiologies are always associated with the initiation and progression of hepatic fibrosis^1,27^. p53-upregulated modulator of apoptosis (PUMA), is one of the most potent mediators of apoptosis induced by various stimuli^28^. *PUMA* deficiency could block cell apoptotic responses to p53 activation, DNA-damaging agents, and hypoxia in hepatocytes^29^. In response to multiple insults, PUMA functions through other Bcl-2 family members, including Bcl-2, Mcl-1, and Bcl-xL, to induce mitochondrial dysfunction and caspases activation^30,31^. PUMA-mediated apoptotic response in hepatocytes is a direct cause of compensatory proliferation and carcinogenesis in the liver^32^. Mice deficient in *NF-κB* signaling or the anti-apoptotic Bcl-2 family members developed spontaneous hepatocellular carcinoma secondary to hepatocytes death^33^, and PUMA has been demonstrated to be a target of NF-κBp65 and a critical mediator of TNF-α-induced hepatocytes apoptosis^34^. However, the role of NF-κBp65 in PUMA-regulated hepatocytes apoptosis in liver fibrogenesis remains unknown.

In the current study, we found that Fas/FasL promoted PUMA-mediated hepatocytes apoptosis via autophagy and NF-κBp65 signaling, while inhibition of autophagy or *PUMA* deficiency attenuated Fas/FasL-modulated hepatocytes apoptosis and liver fibrosis. Furthermore, hepatocytes deletion of *NF-κBp65* promoted PUMA-mediated hepatocytes apoptosis via regulating the Bcl-2 family, and the enhanced hepatocytes apoptosis linked inflammatory action to drive HSCs activation and liver fibrogenesis. Thus, we suggest that Fas/FasL contributes to NF-κBp65/PUMA-regulated hepatocytes apoptosis via autophagy to enhance liver fibrogenesis.

## Results

### Fas/FasL-mediated apoptosis involved in liver fibrogenesis

**Fig. 1:**
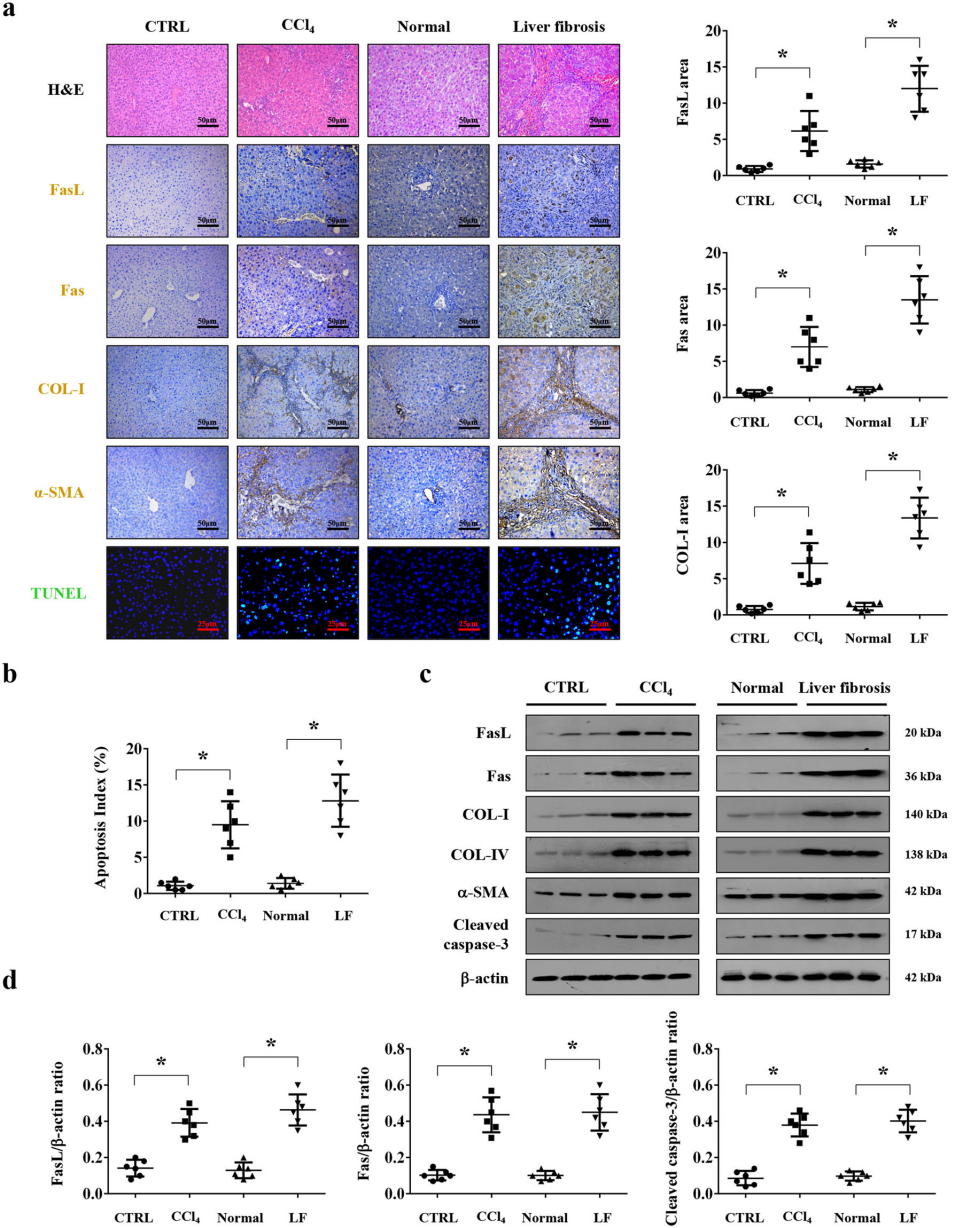
Fas/FasL-mediated apoptosis involved in liver fibrogenesis. **a** H&E staining, Fas, FasL, collagen-I (COL-I), α-SMA immunohistochemical staining (brown), and TUNEL staining (green) in the related liver tissues were presented. FasL, Fas, and COL-I area from the histological staining were also determined. Values are presented as mean ± SEM. *P < 0.05, *n* = 6 per group. CTRL, the control mice (olive oil-treated mice); CCl_4_, 20% carbon tetrachloride-induced mouse fibrosis; Normal, healthy volunteers; LF, human liver fibrosis. **b** The apoptotic index from TUNEL staining was presented. *n* = 6 in each group, values are presented as mean ± SEM. *P < 0.05. **c** Western blotting represented that the levels of Fas, FasL, α-SMA, COL-I, COL-IV, and cleaved caspase-3 were enhanced in liver fibrotic sections. β-actin was used as the loading control. **d** The ratio of densitometry units of the normalized FasL/β-actin, Fas/β-actin, and cleaved caspase-3/β-actin was also presented (*n* = 6 per group), values are presented as mean ± SEM. *P < 0.05.

To evaluate the role of Fas/FasL in liver fibrosis, liver specimens from healthy volunteers and liver fibrosis patients were analyzed. Histological staining presented a loss of preserved architecture and excess deposition of ECM, associated with obvious hepatic apoptosis and upregulated Fas/FasL expression, were observed in the liver fibrotic tissues compared with the normal samples (Fig. 1a, b). Western blotting also confirmed a similar condition (Fig. 1c, d). By using mouse model, we found that CCl_4_ injection-induced prominent liver fibrosis in mice as shown by α-SMA and COL-I staining, which was accompanied with obvious apoptosis and Fas/FasL upregulation (Fig. 1a, b), and western blotting further represented that the levels of Fas, FasL, α-SMA, COL-I, COL-IV, and cleaved caspase-3 were enhanced in the fibrotic sections (Fig. 1c, d). These results indicated that Fas/FasL involved in hepatic apoptosis in liver fibrogenesis.

### Autophagy participated in Fas/FasL-mediated hepatic apoptosis in liver fibrosis

**Fig. 2:**
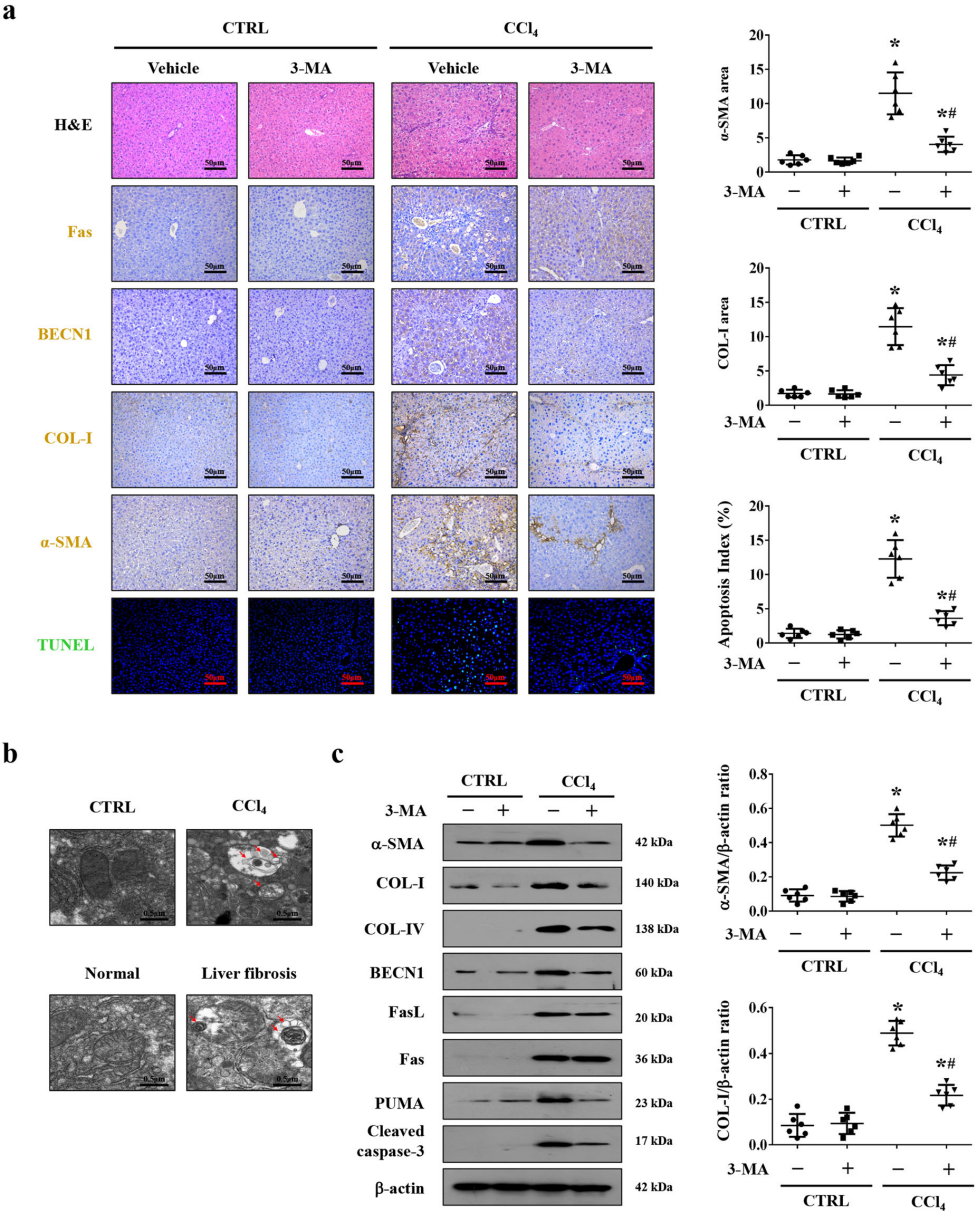
Autophagy participated in Fas/FasL-mediated hepatic apoptosis in liver fibrosis. **a** H&E staining, Fas, BECN1, COL-I, α-SMA staining (brown), and TUNEL staining (green) were presented in the indicated sections from olive oil- (as CTRL) or CCl_4_-treated mice, with or without 3-MA administration. α-SMA area and COL-I area from the histological staining, and the apoptotic index (TUNEL staining) were also determined. *P < 0.05 versus CTRL mice, ^#^*P* < 0.05 versus CCl_4_-treated mice without 3-MA treatment, *n* = 6 per group. **b** Ultrastructural features in the hepatocytes of liver tissues from human tissues and mouse models were presented (red arrows indicating autophagosomes). **c** Western blotting depicted that inhibition of autophagy by 3-MA downregulated the expressions of BECN1, α-SMA, COL-I, COL-IV, PUMA, and cleaved caspase-3, without affecting the state of Fas and FasL, in CCl_4_-treated mice. The ratio of densitometry units of the normalized α-SMA/β-actin and COL-I/β-actin was presented (*n* = 6 per group), values are presented as mean ± SEM. *P < 0.05 versus CTRL mice, ^#^*P* < 0.05 versus CCl_4_-treated mice without 3-MA treatment.

Our preceding data has testified that autophagy was required for liver fibrosis^21^. In the current study, we found that the autophagic element BECN1 was enhanced in liver fibrosis, which was associated with the upregulated α-SMA and COL-I expressions and apoptotic signaling (Fig. 2a), and ultrastructural analysis showed the typical autophagosomes in the hepatocytes of mouse and human liver fibrotic sections but scarce in the normal tissues (Fig. 2b). We pretreated mice with autophagic inhibitor 3-methyladenine (3-MA) and found 3-MA treatment alleviated the degree of apoptosis and liver fibrosis in mice, and did not affect the levels of Fas and FasL (Fig. 2a, c and Supplementary Fig. 1a). To sum up, these data suggested that autophagy participated in Fas/FasL-mediated hepatic apoptosis in liver fibrosis.

### PUMA responded to Fas/FasL/autophagy-regulated hepatocytes apoptosis and HSCs activation during liver fibrosis

**Fig. 3:**
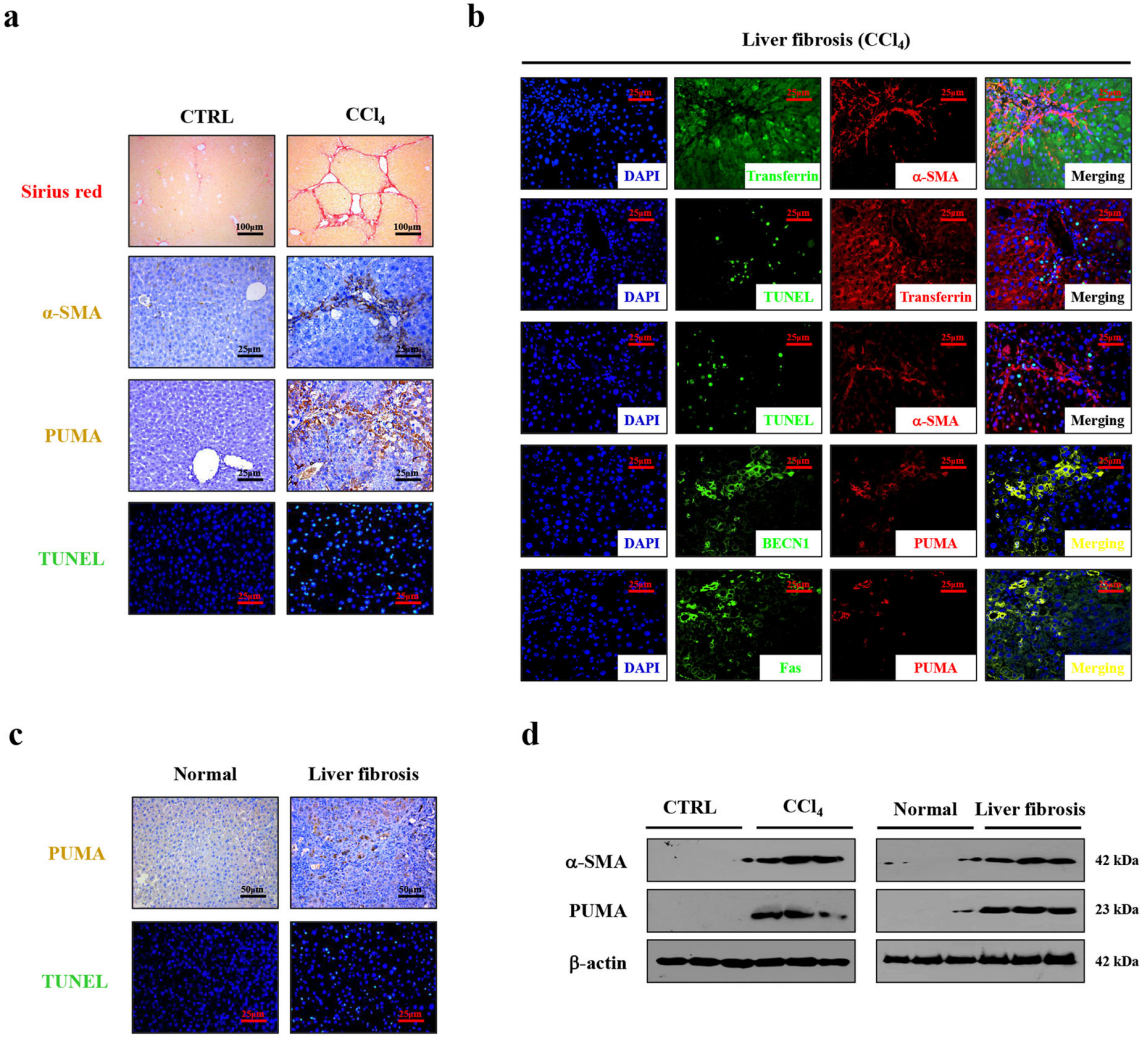
PUMA responded to Fas/FasL/autophagy-mediated hepatocytes apoptosis during liver fibrosis. **a** Sirius red staining (red), α-SMA (brown), PUMA staining (brown), and TUNEL staining (green) were presented in the indicated sections from CTRL and CCl_4_-treated mice (*n* = 6 per group). **b** Double IF staining by utilizing HSCs marker α-SMA (red) and hepatocytes marker transferrin (green) were presented (upper panel). Double staining of transferrin (red) and TUNEL (green), α-SMA (red), and TUNEL (green) indicated that the apoptotic cells mainly located in hepatocytes in liver fibrotic mice (middle panel). Co-staining of BECN1 (green) and PUMA (red), Fas (green), and PUMA (red) further revealed that PUMA involved in Fas and autophagy-regulated signaling (lower panel). Nuclei (blue) were counterstained with DAPI (4′6-diamidino-2-phenylindole dihydrochloride). **c** PUMA immunohistochemistry staining (brown) and TUNEL staining (green) in the indicated humans liver sections were presented. **d** PUMA and α-SMA levels were determined by western blotting.

PUMA has been demonstrated to be a target of Fas/FasL signaling and a critical mediator of apoptosis in our previous study^35^. By analyzing the mouse models, we found that, accompanying by the loss of preserved architecture and excess production and deposition of ECM, the expressions of α-SMA, PUMA and the number of apoptotic cells (TUNEL staining) were increased in the liver fibrotic tissues (Fig. 3a). The enhanced PUMA expression and hepatic apoptosis were also observed in the human fibrotic tissues (Fig. 3c). Western blotting further revealed that the levels of α-SMA and PUMA were obviously enhanced in the mouse and human liver fibrotic tissues (Fig. 3d). Double staining by utilizing HSCs marker α-SMA and hepatocytes marker transferrin revealed the apoptotic cells were mainly localized in hepatocytes but not in HSCs (Fig. 3b). Co-staining of BECN1 and PUMA, Fas, and PUMA demonstrated they were mainly localized in similar cells (Fig. 3b). Western blotting showed that inhibition of autophagy by 3-MA repressed the upregulation of PUMA and caspase-3 cleavage in CCl_4_-induced mouse fibrosis (Fig. 2c and Supplementary Fig. 1a).

**Fig. 4:**
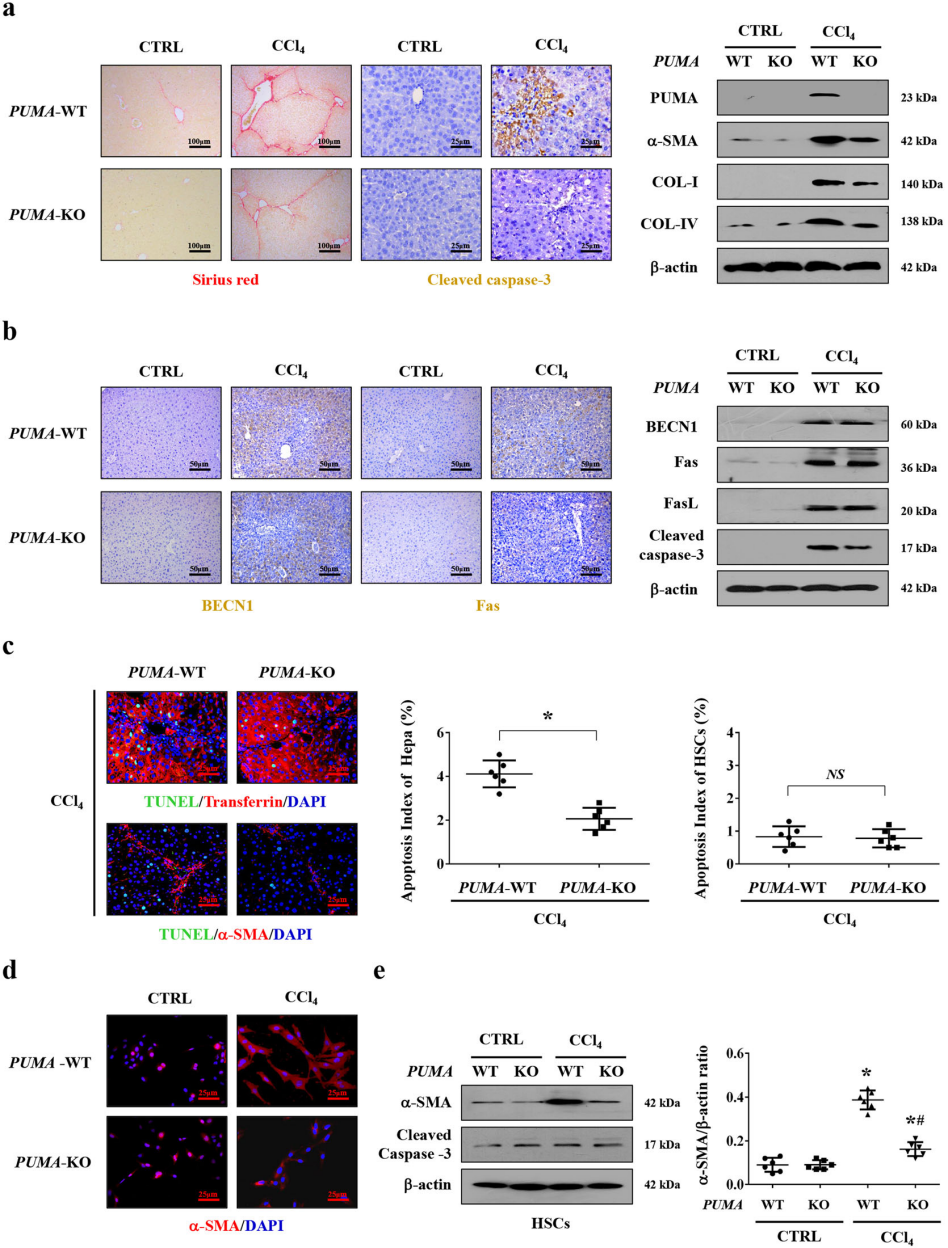
Targeted deletion of *PUMA* ameliorated hepatocytes apoptosis and liver fibrosis. **a** Sirius red staining (red) and cleaved caspase-3 staining (brown) presented that targeted deletion of *PUMA* ameliorated hepatic apoptosis and collagen deposition. Western blotting presented that targeted deletion of *PUMA* ameliorated the levels of collagen-IV (COL-IV), collagen-I (COL-I), and α-SMA in CCl_4_-treated mice (*n* = 6 per group). **b** Immunohistochemistry staining and western blotting revealed that *PUMA* deficiency did not affect the status of Fas and BECN1 in CCl_4_-treated mice. **c** Double immunofluorescence staining (transferrin (red) and TUNEL (green), α-SMA (red) and TUNEL (green)) and the analysis of the apoptotic index of hepatocytes or HSCs indicated that targeted deletion of *PUMA* mainly ameliorated hepatocytes apoptosis during liver fibrosis, *n* = 6 per group. Nuclei (blue) were counterstained with DAPI. *P < 0.05. *NS*, no significance. **d** α-SMA (red) staining in the primary HSCs dissociated from the indicated *PUMA*-WT and *PUMA*-KO mice was represented, nuclei (blue) were counterstained with DAPI. **e** The indicated proteins from primary HSCs analyzed by western blotting. The ratio of densitometry units of the normalized α-SMA/β-actin was also determined, *n* = 6 per group, values are presented as mean ± SEM. *P < 0.05 versus primary HSCs from CTRL mice, ^#^*P* < 0.05 versus primary HSCs from CCl_4_-treated *PUMA*-WT mice.

CCl_4_-induced *PUMA*-WT and *PUMA*-KO liver fibrotic mouse models were adopted, and we found that deletion of *PUMA* ameliorated the collagen deposition, the levels of COL-IV, COL-I, α-SMA, and caspase-3 cleavage in CCl_4_-treated mice, although there was no obvious distinction between *PUMA*-WT and *PUMA*-KO mice from the control group (Fig. 4a). By the way, PUMA was reported to be expressed lowly or barely in normal cells and tissues, but is rapidly induced in response to a wide range of stresses in human and mouse cells^29,31^. Due to a very low level of PUMA in normal tissues, the low concentration of total detected proteins, and the limited detection capacity, we could not detect obvious PUMA signaling in the normal *PUMA*-WT mouse and further make a definite distinction between normal *PUMA*-WT mouse and normal *PUMA*-KO mouse, however, the levels of PUMA were obviously enhanced in the mouse and human liver fibrotic sections (Fig. 3a, c, d and Fig. 4a). *PUMA* deficiency did not affect the levels of Fas, FasL, and BECN1, but downregulated cleaved caspase-3 in CCl_4_-induced mouse fibrosis (Fig. 4b). By utilizing co-staining analysis, we found the apoptotic cells were mainly localized in hepatocytes, and deletion of *PUMA* decreased the number of hepatocytes apoptosis (Fig. 4c). Lastly, the activation of primary HSCs dissociated from CCl_4_-treated *PUMA*-WT mice was enhanced compared with that from CCl_4_-treated *PUMA*-KO mice, while *PUMA* deletion could not interfere with the apoptosis of primary HSCs (Fig. 4d, e). These observations revealed that PUMA contributed to Fas/FasL/autophagy-regulated hepatocytes apoptosis and HSCs activation in liver fibrosis.

### Fas/FasL repressed the activation of NF-κBp65 in hepatocytes in liver fibrogenesis

**Fig. 5:**
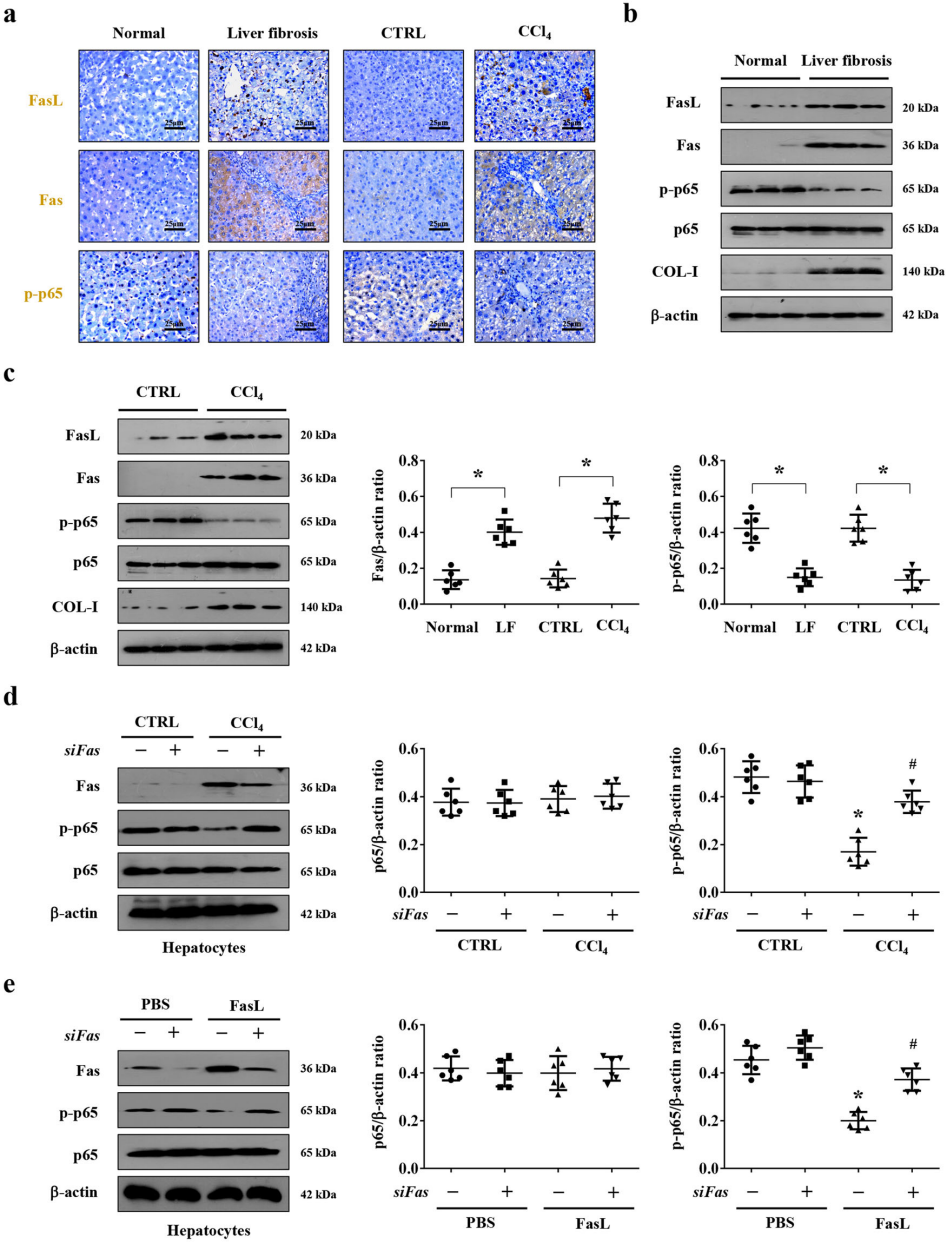
Fas/FasL repressed the activation of NF-κBp65 in hepatocytes in liver fibrogenesis. **a** Immunohistochemical staining (brown) of Fas, FasL, and p-p65 (the phosphorylation of NF-κBp65) in the indicated liver sections was presented (*n* = 6 per group). **b** Expressions of Fas, FasL, p65, p-p65, and COL-I proteins from the related humans liver sections were detected by western blotting. **c** Western blotting was adopted to analyze the levels of Fas, FasL, p65, p-p65, and COL-I in the liver tissues of mouse models. The ratio of densitometry units of the normalized Fas/β-actin and p-p65/β-actin from (**b**) and (**c**) was also determined, *n* = 6 per group, values are presented as mean ± SEM. *P < 0.05. CTRL, the control mice (olive oil-treated mice); CCl_4_, 20% carbon tetrachloride-induced mouse fibrosis; Normal, healthy volunteers; LF, human liver fibrosis. **d** Western blotting depicted that knockdown of *Fas* upregulated the level of p-p65 in the primary hepatocytes isolated from CCl_4_-treated mice. The ratio of densitometry units of the normalized p65/β-actin and p-p65/β-actin was also presented, *n* = 6 per group, values are presented as mean ± SEM. *P < 0.05 versus primary hepatocytes from CTRL group, ^#^*P* < 0.05 versus primary hepatocytes from CCl_4_-treated group without *siFas* treatment. **e** FasL treatment enhanced the expression of Fas and repressed the phosphorylation of NF-κBp65 (p-p65) in the primary hepatocytes, while knockdown of *Fas* by siRNA upregulated the level of p-p65 in FasL-treated group. The ratio of densitometry units of the normalized p65/β-actin and p-p65/β-actin was also presented, *n* = 6 per group, values are presented as mean ± SEM. *P < 0.05 versus primary hepatocytes from PBS group, ^#^*P* < 0.05 versus primary hepatocytes from FasL-treated group without *siFas* treatment.

Decreased or absent NF-κB activity in hepatocytes might lead to subsequent fibrosis by regulating hepatocytes injury and the primary trigger of fibrogenic responses in the liver, and NF-κBp65 involved in the regulation of various pathological events in chronic liver diseases^9^. To investigate the correlation between Fas/FasL and NF-κBp65 in liver fibrosis, liver specimens from humans and CCl_4_-induced mouse model were analyzed, and histological staining presented that, in contrast to the upregulation of Fas/FasL signaling in the fibrotic tissues, the activation of NF-κBp65 (phosphorylation of NF-κBp65, p-p65) was obviously repressed in the liver fibrotic tissues compared with their normal samples (Fig. 5a). Western blotting also confirmed a similar condition (Fig. 5b, c). By using primary hepatocytes isolated from the mouse models, upregulated Fas expression and downregulated p-p65 level were observed in the primary hepatocytes dissociated from CCl_4_-treated mice, while knockdown of *Fas* by siRNA significantly enhanced the activation of NF-κBp65 (p-p65) in the primary hepatocytes dissociated from CCl_4_-treated mice (Fig. 5d). What is more, FasL administration promoted the expression of Fas and repressed the level of p-p65 in the primary isolated hepatocytes cells, and knockdown of *Fas* promoted the activation of NF-κBp65 (Fig. 5e). By the way, we also found that knockdown of *Fas* did not obviously influence the status of NF-κBp65 and its phosphorylation in the control group, which might suggest that a very low level of Fas/FasL signaling under normal condition could not regulate NF-κBp65 pathway in hepatocytes. In summary, these findings indicated that Fas/FasL repressed the activation of NF-κBp65 in hepatocytes in liver fibrogenesis.

### NF-κBp65 inhibited PUMA-mediated hepatocytes apoptosis via Bcl-2 family and attenuated liver fibrosis

**Fig. 6:**
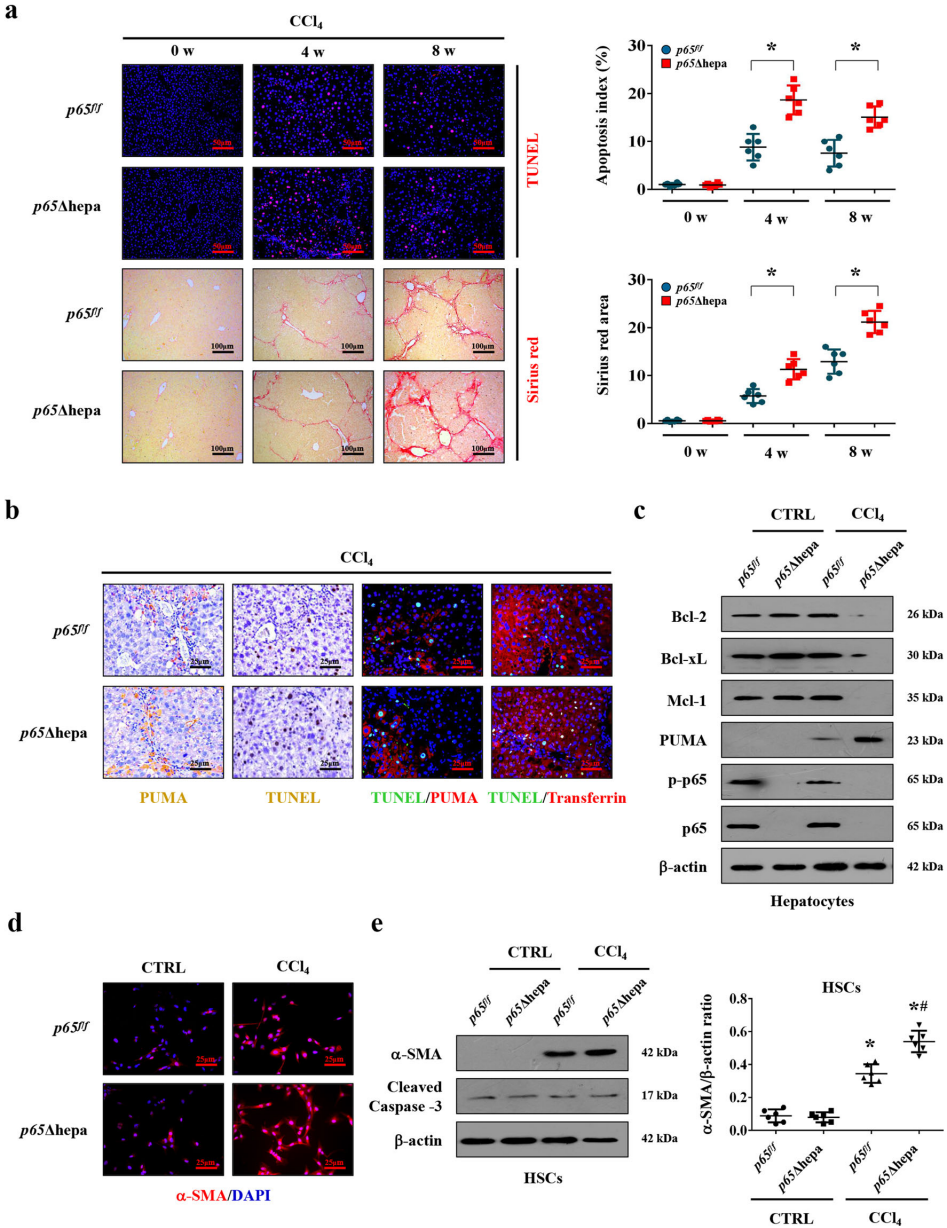
NF-κBp65 inhibited PUMA-mediated hepatocytes apoptosis and attenuated liver fibrosis. **a** TUENL staining (red) and sirius red staining at the indicated time points were adopted to showed that *NF-κBp65* deficiency in hepatocytes promoted apoptosis and collagen deposition during fibrogenesis. The apoptotic index and sirius red area were also analyzed. *P < 0.05. *p65*Δhepa: hepatocytes specific *NF-κBp65* deletion; *p65*^*f/f*^: *NF-κBp65* wild-type. **b** Immunohistochemical staining revealed *NF-κBp65* deficiency in hepatocytes induced PUMA expression and cell apoptosis (brown). Double staining of TUNEL (green) and PUMA (red) indicated that PUMA expression and TUNEL signaling were located in similar cells, and co-staining of TUNEL (green) and transferrin (red) was also presented. Cell nuclei (blue) were counterstained by DAPI. **c** Western blotting represented hepatocytes specific *NF-κBp65* deletion promoted the upregulation of PUMA and downregulated the levels of anti-apoptotic proteins Bcl-2, Bcl-xL, and Mcl-1 in primary hepatocytes isolated from CCl_4_-treated mice, and the phosphorylation of NF-κBp65 (p-p65) was repressed in primary hepatocytes isolated from CCl_4_-treated *p65*^*f/f*^ mice. **d** Primary HSCs dissociated from the indicated mice were stained by α-SMA (red). Nuclei (blue) were counterstained with DAPI. **e** The indicated proteins from primary isolated HSCs in the mouse models were detected by western blotting. The ratio of densitometry units of the normalized α-SMA/β-actin was also determined. *P < 0.05 versus primary HSCs from CTRL mice, ^#^*P* < 0.05 versus HSCs from CCl_4_-treated *p65*^*f/f*^ mice. *n* = 6 per group.

By using hepatocytes specific *NF-κBp65* deletion (*p65*Δhepa) and *NF-κBp65* wild-type (*p65*^*f/f*^) fibrotic mouse models, TUNEL analysis performed at the different time-points of fibrosis showed that cells apoptosis was exacerbated in CCl_4_-treated *p65*Δhepa mice, with an apoptotic index that was higher compared with *p65*^*f/f*^ mice. What is more, sirius red staining detected at the distinct points of fibrosis development disclosed that significant enhanced accumulation of collagen was found in CCl_4_-treated *p65*Δhepa mice than CCl_4_-treated *p65*^*f/f*^ mice (Fig. 6a). Moreover, the phosphorylation of NF-κBp65 (p-p65) was repressed in CCl_4_-treated *p65*^*f/f*^ mice, and p-p65-positive signaling in *p65*Δhepa mice was only localized in the non-parenchymal cells due to hepatocytes specific *NF-κBp65* deletion in *p65*Δhepa mice (Supplementary Fig. 2a), these data suggest that downregulated or absent NF-κBp65 activity could lead to increase hepatocytes apoptosis in the initiation of liver fibrosis. The levels of PUMA, associates with cell apoptosis, induced by CCl_4_ were upregulated in the liver tissues of *p65*Δhepa mice compared to *p65*^*f/f*^ mice (Fig. 6b, c). Double staining of TUNEL and PUMA, TUNEL, and transferrin indicated that PUMA and TUNEL signaling were mainly located in hepatocytes of CCl_4_-treated *p65*Δhepa mice (Fig. 6b). Furthermore, we found that the activation (detected by α-SMA) of primary HSCs rather than their death (detected by cleaved caspase-3) was visibly enhanced in CCl_4_-treated *p65*Δhepa mice than that in *p65*^*f/f*^ mice (Fig. 6d, e).

The pro-survival Bcl-2 family members actively sequester the pro-apoptotic BH3-only members, like PUMA, and finally inhibit the induction of apoptosis^36–38^. By microanalysis screening for primary hepatocytes from mouse models, we found that *PUMA* (*BBC3*) in primary hepatocytes from fibrotic tissues presented a higher expression than that from normal tissues, while the Bcl-2 family members were not affected, it seems that the unchanged Bcl-2 family could not be enough to suppress the detrimental effects of PUMA during liver fibrosis (Supplementary Fig. 2b). Western blotting revealed that the expressions of Bcl-2, Bcl-xL, and Mcl-1 were downregulated in the primary hepatocytes of *p65*Δhepa fibrotic mice (Fig. 6c). Adopting the Bcl-2 inhibitor ABT-199, we revealed that the expressions of PUMA and α-SMA, along with enhanced hepatocytes apoptosis and collagen deposition, were upregulated compared to that without ABT-199 administration (Supplementary Fig. 2c). By the way, the phosphorylation of NF-κBp65 (p-p65) was repressed in primary hepatocytes isolated from CCl_4_-treated *p65*^*f/f*^ mice compared to those from the control group (Fig. 6c), while ABT-199 did not influence the phosphorylation of NF-κBp65 in primary hepatocytes both from the control group and CCl_4_-treated *p65*^*f/f*^ mice (Supplementary Fig. 2d). Lastly, primary HSCs analysis suggested that ABT-199 administration promoted HSCs activation in liver fibrosis (Supplementary Fig. 2e), These data suggested that NF-κBp65 regulated PUMA-mediated hepatocytes apoptosis and liver fibrosis via Bcl-2 family.

### NF-κBp65/PUMA-regulated hepatocytes apoptosis drove inflammatory response to promote HSCs activation and liver fibrosis

**Fig. 7:**
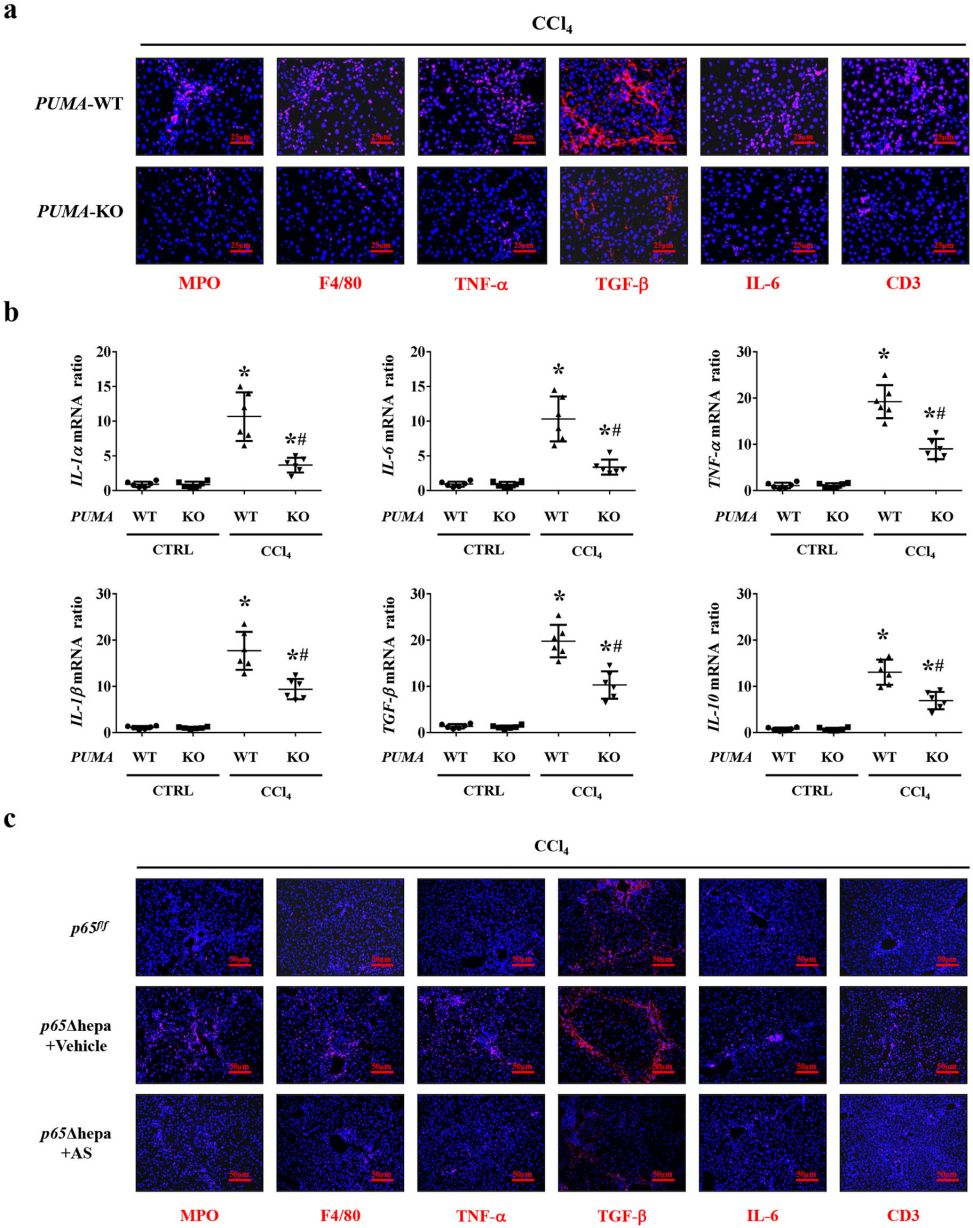
NF-κBp65/PUMA-regulated hepatocytes apoptosis enhanced hepatic inflammatory response. **a** The indicated inflammatory cytokines in the mouse livers were analyzed via immunofluorescence staining (red), the related cytokines were significantly repressed in CCl_4_-treated *PUMA*-KO mice, nuclei (blue) were counterstained with DAPI. **b** Inflammatory factors mRNA expression in the livers from either CCl_4_-treated *PUMA*-WT or *PUMA*-KO mice were analyzed by quantitative reverse-transcription PCR. *P < 0.05 versus CTRL mice, ^#^*P* < 0.05 versus CCl_4_-treated *PUMA*-WT mice. The expression of *β-actin* in each tissue was quantified as the internal control. *n* = 6 per group. **c** Expressions of indicated inflammatory cytokines in the livers were analyzed via immunofluorescence staining (red), the related cytokines were repressed in CCl_4_-treated *p65Δhepa* mice following PUMA antisense oligonucleotides (AS), nuclei (blue) were counterstained with DAPI.

**Fig. 8:**
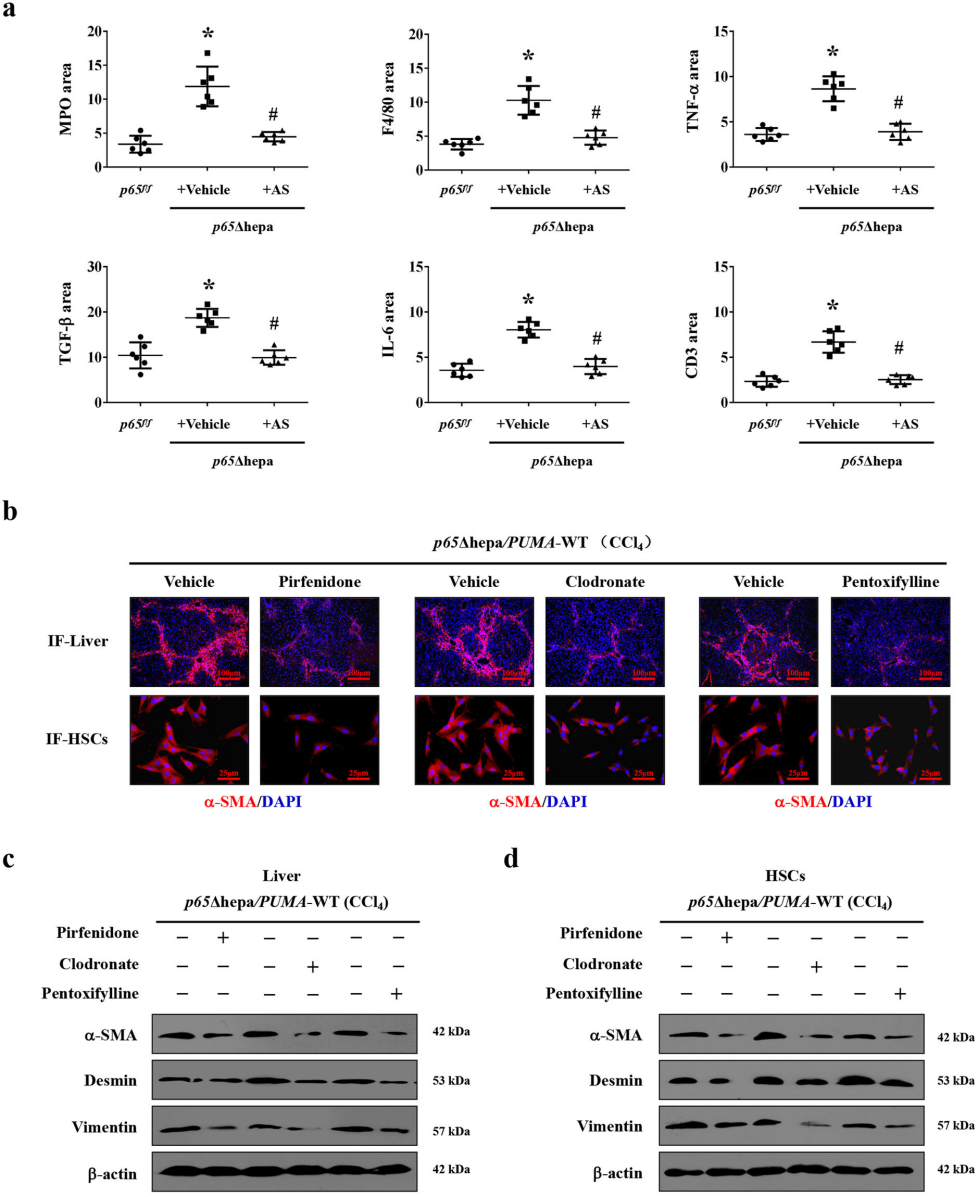
NF-κBp65/PUMA-regulated hepatocytes apoptosis-linked inflammatory response to promote HSCs activation and liver fibrosis. **a** The area of the indicated inflammatory cytokines from immunofluorescence staining of Fig. 7c was analyzed. *P < 0.05 versus *p65*^*f/f*^ mice, ^#^*P* < 0.05 versus *p65*Δhepa mice with vehicle administration. **b** The administration of TGF-β1 inhibitor pirfenidone, TNF-α inhibitor pentoxifylline or monocytes inactivator clodronate-loaded liposomes, attenuated HSCs activation (α-SMA staining of primary HSCs, red) and liver fibrosis (α-SMA staining, red) in *p65*Δhepa/*PUMA*-WT mice. Nuclei (blue) were counterstained with DAPI, *n* = 6 per group. IF, immunofluorescence staining. **c**, **d** Western blotting presented that pirfenidone, pentoxifylline, or clodronate-loaded liposomes attenuated liver fibrosis (liver section) and the activation of HSCs (primary HSCs section) in CCl_4_-induced *p65*Δhepa/*PUMA*-WT mouse model, respectively. *n* = 6 per group.

Dying hepatocytes could release alarmins and activate inflammatory cells to produce various inflammatory cytokines to contribute to the initiation and development of liver fibrosis^9,10^. Based on these, after CCl_4_ treatment, the hepatic inflammatory cells or mediators, including myeloperoxidase (MPO, neutrophil), F4/80 (macrophages), TNF-α, TGF-β, IL-6, and CD3 (lymphocytes) were upregulated in *PUMA*-WT mice compared to *PUMA*-KO mice (Fig. 7a). The inflammatory cytokines mRNA, including *IL-1α*, *IL-6*, *TNF-α*, *IL-1β*, *TGF-β,* and *IL-10*, were also enhanced in CCl_4_-treated *PUMA*-WT mice (Fig. 7b). Furthermore, MPO, F4/80, TNF-α, TGF-β, IL-6, and CD3 were upregulated in *p65*Δhepa mice compared to *p65*^*f/f*^ mice, while PUMA antisense oligonucleotides (AS) treatment suppressed this inflammatory response (Figs. 7c and 8a). By adopting the TGF-β1 inhibitor pirfenidone, TNF-α inhibitor pentoxifylline, and monocytes depressor clodronate-loaded liposomes in *p65*Δhepa/*PUMA*-WT mice, we found inhibition of inflammatory action ameliorated liver fibrosis (Fig. 8b, c). α-SMA levels in primary HSCs isolated from the CCl_4_-treated *p65*Δhepa/*PUMA*-WT mice were visibly upregulated, while above inhibitors administration could hold back the activation of HSCs (Fig. 8b, d). These data indicated that PUMA-promoted HSCs activation depended on the inflammatory response following hepatocytes apoptosis.

**Fig. 9:**
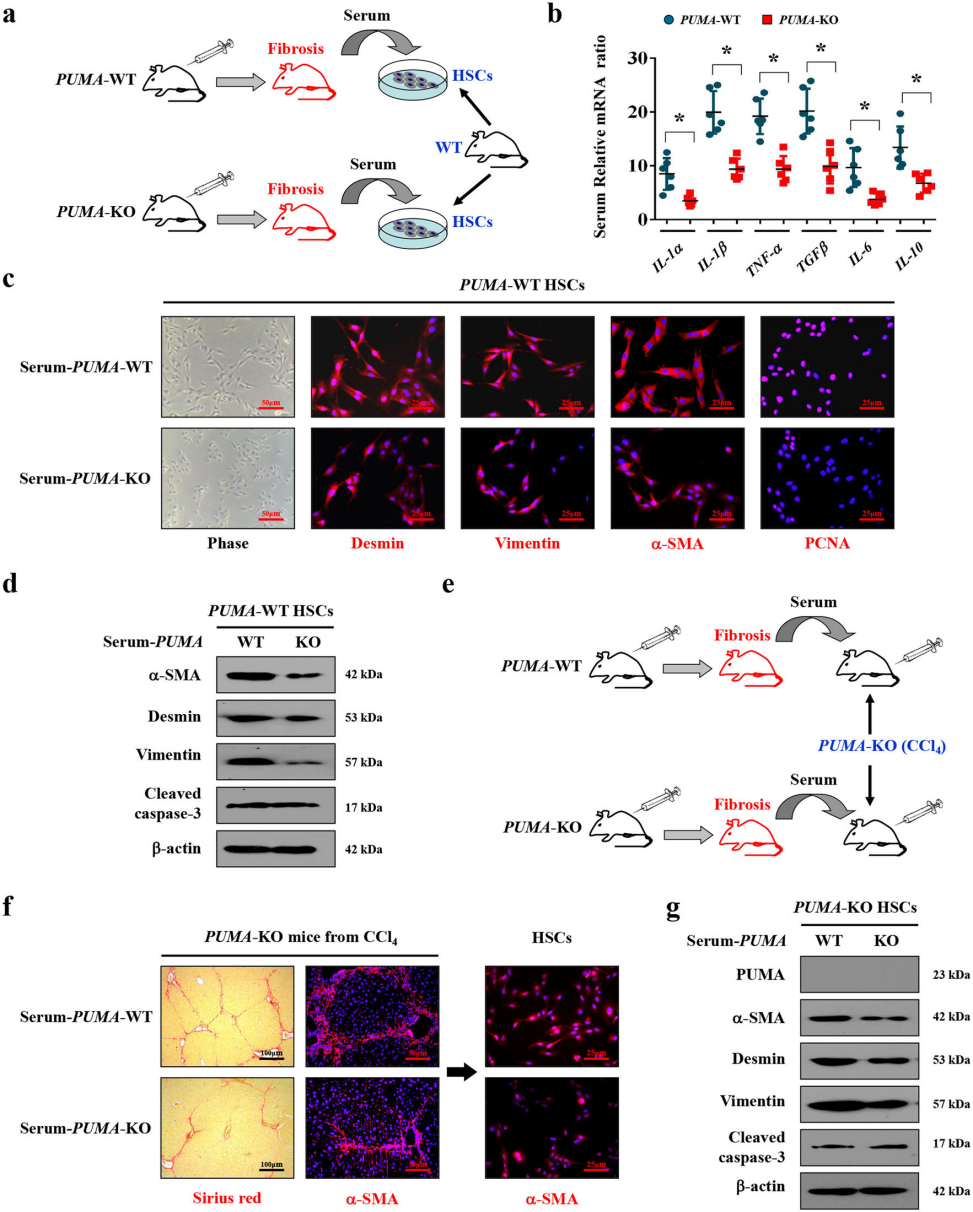
NF-κBp65/PUMA-driven liver inflammation-induced HSCs activation and liver fibrosis. **a** Schematic diagram of the serum test in vitro. **b** The genes expressions of the indicated inflammatory cytokines from CCl_4_-treated *PUMA*-WT or *PUMA*-KO mice were detected by quantitative reverse-transcription PCR. The expression of *β-*actin in each tissue was quantified as the internal control. *n* = 6 per group. *P < 0.05. **c** Representative images of the growth with activation of primary isolated HSCs following the treatment of the distinct serum extracted from CCl_4_-treated *PUMA*-WT or *PUMA*-KO mice. Nuclei (blue) were counterstained with DAPI. **d** Expressions of the related proteins of primary HSCs after serum treatment were detected by western blotting, revealing that *PUMA*-WT serum enhanced the activation of HSCs without affecting cell apoptosis. *n* = 6 per group. **e** Schematic diagram of the serum test in vivo. **f** Sirius red staining (red) and α-SMA staining (red) were examined in the liver tissues of *PUMA*-KO mice following the treatment of the serum extracted either from CCl_4_-treated *PUMA*-WT or *PUMA*-KO mice. The primary isolated HSCs were also analyzed by α-SMA staining (red). Nuclei (blue) were counterstained with DAPI. **g** The indicated proteins of the primary isolated HSCs from *PUMA*-KO mice after serum treatment were detected by western blotting, *n* = 6 per group.

Following the induction of liver fibrosis in CCl_4_-induced *PUMA*-WT and *PUMA*-KO mouse models, these mice serum was extracted respectively (Fig. 9a). The inflammatory mediators mRNA levels from *PUMA*-WT mice, including *IL-1α*, *IL-6*, *TNF-α*, *IL-1β*, *TGF-β,* and *IL-10*, were higher than that from *PUMA*-KO mice (Fig. 9b). The primary HSCs (isolated from normal *PUMA*-WT mice) accepted the serum of *PUMA*-WT liver fibrotic mice showed enhanced expressions of desmin, vimentin, α-SMA, and PCNA (proliferating cell nuclear antigen), in contrast to the HSCs accepting *PUMA*-KO mice serum (Fig. 9c). Similar changes in the HSCs activation and growth elements were also observed by western blotting analysis (Fig. 9d). By in vivo serum test, the serum was extracted from CCl_4_-induced liver fibrosis models in *PUMA*-WT and *PUMA*-KO mice, respectively, and then was transfused to the different *PUMA*-KO littermates via tail intravenous injection (Fig. 9e). Following that, mice who accepted *PUMA*-WT serum appeared increased collagen deposition and enhanced HSCs activation rather than apoptosis, compared to that accepted *PUMA*-KO serum (Fig. 9f, g). To sum up, these data demonstrated that NF-κBp65/PUMA-regulated hepatocytes apoptosis drove inflammatory response to promote HSCs activation and liver fibrosis.

## Discussion

Hepatocytes apoptosis has been considered to be in a series of biochemical steps in most forms of liver injury under a number of pathological conditions^10^. Regulation of hepatocytes apoptosis is crucial for liver homeostasis and increased sensitivity of hepatocytes towards apoptosis results in chronic liver injury, which is linked to trigger inflammatory and wound healing responses that in the long run promote the development of hepatic fibrosis^10^. So far, three classic pro-apoptotic networks, TNF-α/TNF receptor 1 (TNFR1), Fas/FasL, and TNF-related apoptosis-inducing ligand (TRAIL)/death receptor 4 (DR4 or TRAIL-R1)/death receptor 5 (DR5 or TRAIL-R2), have been widely demonstrated to mediate caspase-dependent cellular apoptosis^39,40^. Our previous research has revealed that autophagic flux involved in liver fibrogenesis^21^. Autophagy has long been recognized as a critical pathway in the regulation of cell death, and the autophagic machinery could directly interface with pro-apoptotic factors pathways to promote cell death^14,21^. Several studies have focused on the role of Fas/FasL signaling during autophagy, and Fas/FasL has been verified to mediate autophagy activation in cell line systems including human neuroblastoma (SH-SY5Y) cells, human lung cancer SPC-A-1 cells and HeLa cells, and Fas signaling could activate autophagic cascades in retina-retinal pigment epithelium (RPE) separation during retinal detachment via regulating the conversion of LC3-I to LC3-II and the expression of Atg5. Furthermore, inhibition of autophagy by 3-MA could obviously reverse the outcome mediated by Fas/FasL signaling^41–43^. They also revealed that Fas/FasL promoted the activation of autophagy via modulating Fas-activated death domain (FADD), Src, the c-Jun N-terminal kinase (JNK) family of stress kinases, BECN1, PI3K, etc^41–43^. Given the critical roles that Fas/FasL signaling and autophagy play in liver diseases, in this study, by analyzing the liver sections from both humans and mice, we uncovered that Fas/FasL presented distinctly higher expression in the fibrotic tissues than in normal liver sections, and Fas/FasL signaling is upstream of autophagy, while inhibition of autophagy ameliorated Fas/FasL-regulated hepatic apoptosis, α-SMA expression, and liver fibrosis, and this data suggested that Fas/FasL-mediated hepatic apoptosis by autophagy in liver fibrogenesis.

Autophagy has been reported to control the timing of mitochondrial permeabilization in apoptosis and selectively regulate PUMA level, while *PUMA* depletion prevents sensitization to apoptosis by autophagy inhibition^44,45^. PUMA transduces death signals primarily to the mitochondrial membrane to lead to caspase activation and ultimately cell death via interaction and inhibition of the anti-apoptotic Bcl-2 repertoire^46^. In our study, we found that, accompanying by the loss of preserved architecture and excess deposition of ECM, PUMA was induced in liver fibrosis, and deletion of *PUMA* attenuated HSCs activation and liver fibrosis via inhibiting hepatocytes apoptosis, without influencing the status of the Fas/FasL signaling and autophagy. Moreover, inhibition of autophagy repressed PUMA upregulation and hepatocytes apoptosis to alleviate liver fibrosis, and we suggested that PUMA contributed to the Fas/FasL/autophagy-regulated hepatocytes apoptosis and HSCs activation in liver fibrosis.

NF-κBp65 has a wide range of functions in different cellular compartments influencing the survival of hepatocytes and the activation of HSCs^47^. Recent study has suggested that *miR-196b-5p*-mediated downregulation of Fas involved in the activation of STAT3 signaling through the NF-κBp65/IL-6 axis, and Fas downregulation could activate NF-κBp65 signaling to promote lung cancer cell growth in non-small cell lung cancer (NSCLC)^48^, which powerfully demonstrated the interaction between Fas/FasL and NF-κBp65. In our study, we also verified the correlation between them and found that the activation of NF-κBp65 (phosphorylation of NF-κBp65, p-p65) was repressed in the liver fibrotic tissues compared with their normal samples. By using primary hepatocytes, we further demonstrated that upregulated Fas and downregulated p-p65 were observed in FasL-treated hepatocytes and in the primary hepatocytes dissociated from CCl_4_-treated mice, while knockdown of *Fas* could significantly reverse that and enhance the activation of NF-κBp65, indicating that Fas/FasL signaling repressed the activation of NF-κBp65 in hepatocytes in liver fibrogenesis. Furthermore, we revealed that deletion of *NF-κBp65* in hepatocytes enhanced the expressions of collagen-IV, collagen-I, and α-SMA, along with the increased number of apoptotic hepatocytes in mice, which finally aggravated liver fibrosis. During this process, downregulated or absent NF-κBp65 activity in hepatocytes, which leads to increased hepatocytes apoptosis, plays an important role in the initiation of liver fibrosis. We then examined the Bcl-2 family, which integrates a number of inter- and intracellular cues to determine whether or not the apoptosis pathway should be activated, and found that the pro-survival Bcl-2 family members were downregulated in hepatocytes with specific deletion of *NF-κBp65*, while the potent pro-apoptotic element PUMA was increased following *NF-κBp65* deficiency in hepatocytes, which contributed to the development of liver fibrosis. By the way, the hepatic fibrosis mouse model was established by CCl_4_ for 4 weeks and 8 weeks, and we found that the mouse obtained the highest apoptosis index at the 4th week, and then the apoptotic cells decreased gradually as time goes on. During this process, liver fibrosis was accelerated progressively by the accumulation of collagen and the formation of septa, with a maximum peak at 8th week. This phenomenon may be related to that hepatocyte apoptosis is a pivotal and initial step in the development of liver fibrosis, and the pathogenesis of liver fibrosis owns different pathophysiological changes (the changes of hepatocyte apoptosis, collagen deposition, etc) in the different stage^6,7,21^, and our further study is needed to determine the definite causes of this phenomenon.

Previous studies have recognized that dying hepatocytes release alarmins that could recruit inflammatory cells to produce inflammatory cytokines to regulate the initiation and development of liver fibrosis^8^. In our study, inflammatory cells and mediators were dramatically upregulated in *NF-κBp65* deletion in hepatocytes in liver fibrosis, while PUMA antisense oligonucleotides or *PUMA* deficiency suppressed that inflammatory response. Some researches have shown that the mechanisms by which apoptosis promotes inflammation relate to the activation of the resident macrophages in the liver, and HSCs undergo a process of activation resulting from hepatocytes apoptosis and inflammation^4,49,50^. Adopting the TGF-β1 inhibitor pirfenidone, TNF-α inhibitor pentoxifylline, and monocytes depressor clodronate-loaded liposomes, we demonstrated that inhibition of inflammatory action ameliorated HSCs activation and liver fibrosis. Moreover, our in vivo and in vitro serum data suggested hepatocytes apoptosis contributed to inflammation-induced HSCs activation. These results indicated that HSCs activation depended on the inflammatory response following hepatocytes apoptosis. However, there is much yet to be learned about the complex interplay between the immune system and the hepatocytes status in the pathophysiological states of the liver.

In summary, our investigation provides evidence that Fas/FasL contributes to NF-κBp65/PUMA-regulated hepatocytes apoptosis via autophagy to enhance HSCs activation and liver fibrosis, and this network could be a therapeutic target for liver fibrosis.

## Materials and methods

### Tissue samples

Six normal liver tissues were from parahemangioma sites of hepatic hemangioma patients and the paired samples of liver fibrosis were obtained from 6 hepatitis B virus (HBV)-infected liver fibrosis patients during operations before any therapeutic intervention. Written informed consent was received from each patient and healthy volunteer prior to inclusion in the study and the acquisition of these samples was approved by the Clinical Research Ethics Committee of the Third Affiliated Hospital of Sun Yat-Sen University.

### Mice and treatments

All animal experiments were approved by the Institutional Animal Ethics Committee of the Third Affiliated Hospital of Sun Yat-Sen University. Eight- to ten-week-old male mice (20–25 g) were used for all experiments. All mice were randomly allocated to each group and to collect and process data for analysis, and the experimenters were blinded towards the treatments or genetic background in all the experiments. LoxP *NF-κBp65* (*RelA*) mice were generated on a C57BL/6 gene background. Hepatocytes specific *NF-κBp65* deletion mice (*p65*Δhepa) were generated by crossing the floxed *p65* mice with *Alb-cre* mice, which shows hepatocyte-specific expression of *Cre* recombinase and *NF-κBp65* ablated solely in hepatocytes but not in non-parenchymal liver cells. Floxed *p65* littermates (*p65*^*f/f*^) were used as the wild-type (WT) mice. *PUMA* wild type (*PUMA*-WT) and *PUMA* knockout (*PUMA*-KO) littermates on a C57BL/6 background were generated from *PUMA* heterozygote mice (Jackson Laboratory, Bar Harbor, ME, USA). *p65*^*f/f*^/*PUMA*-WT mice on a C57BL/6 background generated by crossing *p65*^*f/f*^ and *PUMA*-WT littermates, *p65*Δhepa/*PUMA*-WT mice on a C57BL/6 background were generated from *p65*Δhepa and *PUMA*-WT littermates.

The CCl_4_-induced liver fibrosis model was established via an intraperitoneal injection of 20% CCl_4_ (Sinopharm Chemical Reagent, Shanghai, China) dissolved in an olive oil solution (Sinopharm Chemical Reagent) at 5 ml/kg body weight, twice per week for 8 weeks. The control group was intraperitoneally injected only with olive oil at 5 ml/kg body weight, twice per week for 8 weeks. For inhibition of autophagy, the autophagic inhibitor 3-methyladenine (3-MA, Sigma, St Louis, MO, USA) was given with 3 μl of a 20 mg/ml solution prepared in saline, and mice were injected with 3-MA (10 mg/kg) at 30 min before every CCl_4_ injection, and the vehicle group (as Vehicle) was injected with the same volume of saline. For inhibiting inflammatory response, mice were orally administrated with TGF-β1 inhibitor pirfenidone (Sigma) at 250 mg/kg daily for 8 weeks. To inhibit TNF-α action, pentoxifylline (Sigma) at 200 mg/kg was administered by intraperitoneal injection for 8 weeks to mice at 30 min before every CCl_4_ injection. Clodronate-loaded liposomes (Sigma) were intraperitoneally injected in order to deplete monocytes for three consecutive days per week for 8 weeks in the process of mice models. To inhibited PUMA expression in mice, the *PUMA* sense oligonucleotides (5′-A*G*C*GCCATGGCCCGCGC*A*C*G-3′; *phosphorothioate bonds) and *PUMA* antisense oligonucleotides (*PUMA*-AS, 5′-C*G*T*GCGCGGGCCATGGC*G*C*T-3′) were synthesized by GenePharma (Shanghai, China). The mice were treated with *PUMA* sense or *PUMA* antisense oligo at 25 mg/kg/day via intraperitoneal injection for 8 weeks. By inhibiting the Bcl-2 activity, ABT-199 (Venetoclax, 100 mg/kg/d, Santa Cruz, Santa Cruz, CA, USA) was utilized via oral gavage. Six animals were used in each group for the above studies.

### Primary cells isolation and cells culture

Primary hepatocytes and HSCs were dissociated from the indicated mice by a non-recirculating collagenase perfusion as previously described^21,51^. Liver was perfused through the portal vein in situ successively with Ca^2+^-free HBSS (Hank’s Balanced Salt solution) for 15 min, with 100 ml 0.2% pronase solution, and lastly with 0.2% collagenase Type-IV (Sigma) solution until the liver looked digested and became pale in color. This resulting cell suspension was filtered through a 100 μm pore size mesh nylon filter (Sinopharm Chemical Reagent), and then centrifuged for 10 min at 200 × *g*, the pellet was collected for primary hepatocytes and the supernate for primary HSCs. The primary hepatocytes were cultured in RPMI medium 1640 supplemented with 10% heat-inactivated fetal bovine serum (FBS), 100 units/ml penicillin, and 100 μg/ml streptomycin in a humidified incubator at 37 °C with 5% CO_2_. For HSCs isolation, commercially available 60% Optiprep (Sigma) was added to the final concentration 11.5%. The above supernate was centrifuged at 450 × g for 15 min and then suspended with 0.5 ml HBSS, after being centrifuged at 1400 × *g* for 25 min, HSCs on the top of Optiprep layer were collected and also cultured in RPMI medium 1640. These cells were authenticated and tested and they were not contaminated by mycoplasma. For FasL treatment experiments in vitro, FasL (10 ng/ml, Sigma) was added for 12 h, and siRNA treatment was performed as previously described^29^.

### Serum test

For serum test in vitro, following induction of liver fibrosis in CCl_4_-induced *PUMA*-WT and *PUMA*-KO mouse models, these mice were anesthetized and their serum was extracted, respectively. The above-mentioned different serum was added to the primary HSCs isolated from normal *PUMA*-WT mice (without any treatments), respectively, and then cultured in a humidified incubator at 37 °C with 5% CO_2_ for 36 h. For serum test in vivo, the serum was extracted from CCl_4_-induced liver fibrosis models in *PUMA*-WT and *PUMA*-KO mice, respectively. The above-mentioned distinct serum was then transfused to the different *PUMA*-KO littermates (by the third week of 10% CCl_4_ treatment, at 200 μl per mouse for three consecutive days per week until the 8th week) via tail intravenous injection. Following that, the mice were anesthetized and euthanized and the entire liver was analyzed using histopathological detection.

### Histological staining

Sirius red staining was used for collagen determination. Hematoxylin-esoin (H&E) staining, immunohistochemical (IHC), immunofluorescence (IF), and double IF staining were also performed as previously described^51^, and the semi-quantitative analysis of the histological staining and sirius red staining was analyzed using Image-Pro Plus 6.0. IHC and IF staining were performed by using antibodies for Fas (SAB5700608), FasL (SAB4501538), NF-κBp-p65 (p-p65, SAB4504482), NF-κBp65 (p65, SAB4502615) (all from Sigma), collagen-I (COL-I, ab233080), α-SMA (ab5694), vimentin (ab92547), PUMA (ab9643), transferrin (ab278498), TGF-β (ab215715), MPO (ab208670) (all from Abcam, Cambridge, MA, USA), desmin (sc-23879), BECN1 (sc-48341), IL-6 (sc-28343), F4/80 (sc-52664) (all from Santa Cruz), cleaved caspase-3 (9661), CD3 (86603), TNF-α (11948), PCNA (13110) (all from Cell Signaling Technology, Danvers, MA, USA). TUNEL staining was performed using the In Situ Cell Death Detection Kit (Roche, Basel, Switzerland) according to the manufacturer’s instructions. The apoptotic index was determined by dividing the number of apoptotic cells by the total number of cells in the section in at least 10 randomly selected fields (×200). For transmission electron microscopy, the hepatic tissues were fixed as previously described^21^ and observed with a transmission electron microscope (Hitachi, H-800, Tokyo, Japan), and the images were acquired digitally from a randomly selected pool of six fields.

### Western blotting

The related proteins were analyzed by western blotting using anti-α-SMA (ab5694), -vimentin (ab92547), -PUMA (ab9643), -collagen-IV (COL-IV, ab236640, ab6586), -COL-I (ab233080) (all from Abcam), -Fas (SAB5700608), -FasL (SAB4501538), -NF-κBp-p65 (p-p65, SAB4504482), -NF-κBp65 (p65, SAB4502615), -β-actin (A5441) (all from Sigma), -desmin (sc-23879), -Bcl-xL (sc-8392), -Mcl-1 (sc-74437), -Bcl-2 (sc-7382), -BECN1 (sc-48341) (all from Santa Cruz) and -cleaved caspase-3 (9661) (Cell Signaling Technology). Appropriate horseradish peroxidase conjugated secondary antibodies were used to detect the primary antibody/antigen complexes as previously described^35^, and then was quantified for densitometry analysis. For controlling unwanted sources of variation, the quantitative densitometry results were calculated and normalized to the loading control β-actin densitometry units.

### RNA extraction and PCR assays

Total RNA was extracted using the RNAgents Total RNA Isolation System (Promega, Madison, WI, USA) according to the manufacturer’s instruction. Real-time polymerase chain reaction (PCR) was performed on a Chromo 4 Detector System (MJ Research, Sierra Point, CA, USA) using gene-specific primers and DyNAmo SYBR Green Master Mix (Finnzymes, Finland). Relative mRNA ratio was used to analyze the data, and the indicated mRNA in each tissue was normalized by *β*-*actin* gene (as the internal control) from the same tissue and expressed as fold changes relative to the matched control values.

### Microarray experiment

Primary hepatocytes were dissociated from three normal liver samples with olive oil treatment and three matched pairs of fibrotic tissues with CCl_4_ treatment, respectively. Total RNA was isolated by using TRIzol reagent (Invitrogen, Carlsbad, CA, USA), and the total RNA was amplified, labeled, and purified by Affymetrix WT PLUS Reagent Kit (Affymetrix, Santa Clara, CA, USA) and FL-Ovation cDNA Biotin Module V2 (NuGEN, San Carlos, CA, USA) to obtain the biotin-labeled cDNA. Array hybridization and washing were performed using GeneChip Hybridization, Wash and Stain Kit (Affymetrix) in a Hybridization Oven 645 (Affymetrix) and a Fluidics Station 450, and then the arrays were scanned by Affymetrix GeneChip Scanner 3000 (Affymetrix). Command Console Software (Affymetrix) was used to control the scanner and summarize probe cell intensity data (CEL file generation) with default settings. The array data were analyzed for data summarization, normalization, and quality control using the GeneSpring software V12 (Agilent). The data were Log2 transformed and median centered by genes using the Adjust Data function of CLUSTER 3.0 software and then further analyzed with hierarchical clustering with average linkage. Finally, tree visualization was performed by using Java Treeview (Stanford University School of Medicine, Stanford, CA, USA). The microarray dataset is available in the figshare repository (10.6084/m9.figshare.14443382).

### Statistical analysis

The experimenters were blinded towards the treatments or genetic background in all the experiments. To ensure adequate power to detect a pre-specified effect, the sample size was chosen using the Power Analysis and Sample Size (PASS) software. The sample size was estimated even if no statistical methods were used in animal studies. At least six mice or human sections were adopted in each group. Statistical analysis was performed only for studies where each group size was at least *n* = 6, unless otherwise stated. These data were normal distribution and were presented as mean ± SEM, and data statistical analysis were performed using Student’s two-tailed paired *t*-test or one-way ANOVA (more than two groups of data, single factor) or two-way ANOVA (more than two groups of data, two factors), followed by Bonferroni’s comparison post hoc test and post hoc tests are run only if *F* achieved *P* < 0.05. There was no significant variance inhomogeneity, and the variance was similar between the groups that were being statistically compared. Differences were considered statistically significant at the level of *P* < 0.05.

## Supplementary information

Supplementary Figures

## Data Availability

All data generated or analyzed during this study are included in this published article [and its supplementary information files].
