# Supplementary material for: Fas/FasL mediates NF-κBp65/PUMA-modulated hepatocytes apoptosis via autophagy to drive liver fibrosis
Source: Cell Death Dis. 2021 May 12;12(5):474. doi: 10.1038/s41419-021-03749-x (PMC8115181; doi:10.1038/s41419-021-03749-x)
Supplement: Supplementary file 1 — Supplementary Figures [file 41419_2021_3749_MOESM1_ESM.pdf]

Supplementary Figures

a

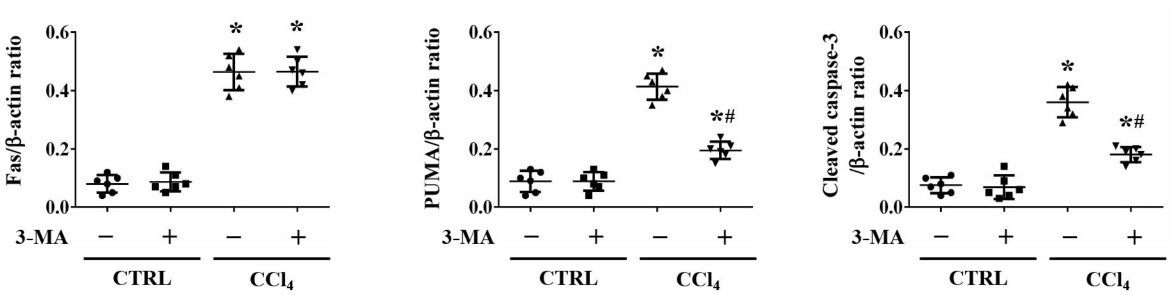

Supplementary Fig. 1 Autophagy participated in Fas/FasL-mediated hepatic apoptosis in liver fibrosis. **a** The ratio of densitometry units of the normalized Fas/β-actin, PUMA/β-actin and cleaved caspase-3/β-actin from Figure **2c** was determined, n = 6 per group, values are presented as mean ± SEM. *P<0.05 versus CTRL mice, #P<0.05 versus CCl_4-treated mice without 3-MA treatment.

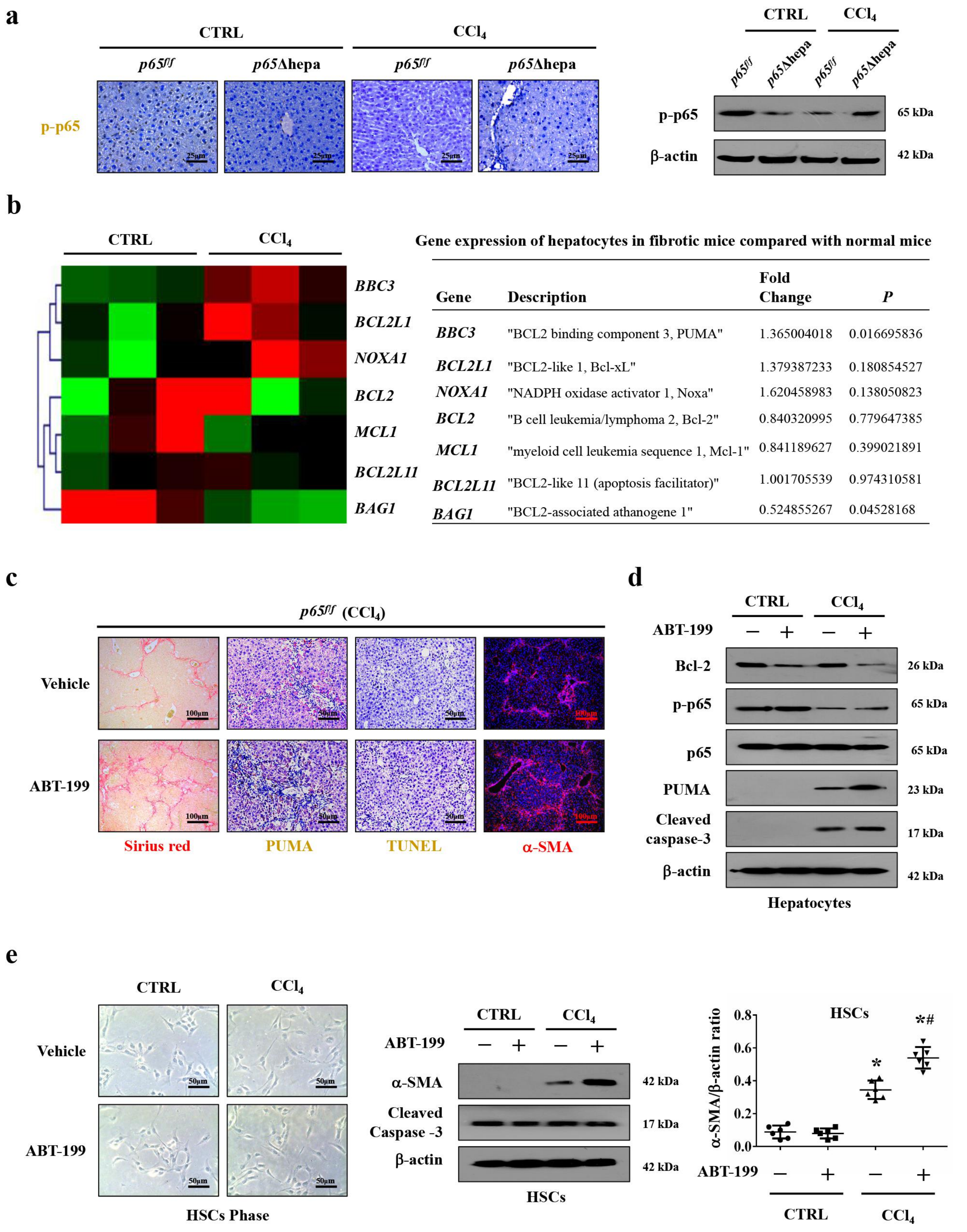

Supplementary Fig. 2 Inhibition of Bcl-2 aggravated NF-κBp65/PUMA-regulated hepatocytes apoptosis and liver fibrosis. **a** The phosphorylation of NF-κBp65 (p-p65, brown) was detected by immunohistochemical (IHC) staining and western blotting in the indicated liver sections. *p65*Δhepa: hepatocytes specific *NF-κBp65* deletion; *p65*$^{f/f}$: *NF-κBp65* wild-type. **b** Two-dimensional

hierarchical clustering results for the Bcl-2 family genes of isolated primary hepatocytes from both of the control mice and CCl_4-treated mice. The fold changes in mRNA levels in fibrotic tissues relative to normal liver tissues were represented by green and red squares, showing decreased and increased levels, respectively. **c** ABT-199, a selective Bcl-2 inhibitor, aggravated collagen deposition (Sirius red staining and α-SMA IF staining), PUMA induction (brown) and cells apoptosis (TUNEL staining, brown) in CCl_4-treated $p65^{f/f}$ mice. **d** Western blotting depicted ABT-199 enhanced the expressions of PUMA and cleaved caspase-3, without affecting the phosphorylation of NF-κBp65 (p-p65), in the primary isolated hepatocytes from CCl_4-treated mice. **e** Representative images of the primary isolated HSCs were shown, and the expressions of α-SMA and cleaved caspase-3 were detected by western blotting. The ratio of densitometry units of the normalized α-SMA/β-actin was also determined. *$P<0.05$ versus HSCs from CTRL mice, #$P<0.05$ versus HSCs from CCl_4-treated mice without ABT-199 administration, $n = 6$ per group.
